# Poland Syndrome Associated with Pernicious Anemia and Gastric Dysplasia

**DOI:** 10.5505/tjh.2012.39259

**Published:** 2012-12-05

**Authors:** Erman Aytaç, Ali Vedat Durgun, Deram Büyüktaş, Deram Büyüktaş, Sibel Erdamar, Şeniz Öngören

**Affiliations:** 1 İstanbul University, Cerrahpasa School of Medicine, Department of General Surgery, İstanbul, Turkey; 2 İstanbul University, Cerrahpasa School of Medicine, Department of Internal Medicine, İstanbul, Turkey; 3 İstanbul University, Cerrahpasa School of Medicine, Department of Pathology, İstanbul, Turkey; 4 İstanbul University, Cerrahpasa School of Medicine, Department of Internal Medicine, Division of Hematology, İstanbul, Turkey

A 55-year-old male with fatigue, dyspnea, and sweating presented to our clinic. Physical examination showed pectus carinatum deformity, flatness on the left side of the chest wall, breast asymmetry, and splenomegaly (Figure 1). Laboratory findings were as follows: macrocytic anemia (red blood cell count: 2x1012/L; hemoglobin: 8.4 g dL–1; hematocrit: 22.2%; mean corpuscular volume: 112 fL [normal range: 80-99]; mean corpuscular hemoglobin: 42 pg [normal range: 27-34]; very low B12 vitamin level: 0 pg mL–1 [normal range: 180-900]; LDH: 918 U L–1 [normal range: 125-243]; total bilirubin: 1.7 mg dL–1 [normal range: 0.2-1.3]). The serum folic acid concentration was normal. Peripheral blood smear showed marked anisocytosis, poikilocytosis, macro-ovalocytes, and hypersegmented neutrophils. Reticulocyte production index was 0.7. Histopathological analysis of a bone marrow biopsy specimen showed hypercellularity, low myeloid:erythroid ratio, and abnormal large RBC precursors with nuclearcytoplasmic asynchrony. Anti-parietal antibodies in the serum were positive at a serum dilution of 1:10. Antiintrinsic antibodies were also positive. Plain chest X-ray showed hyperlucency on the left side of the chest (Figure 2a). Pectus carinatum deformity, agenesis of the left pectoralis major muscle, and bilateral hypertrophy of the sternocleidomastoid muscles were observed via computed tomography (CT) (Figure 2b). 

The patient was diagnosed as PS. Abdominal ultrasonography showed splenomegaly. Vitamin B-12 treatment was initiated (1000 μg d^-^1 for 5 days, 1000 μg week^-^1 for 4 weeks, and then 1000 μg month^-^1 for life). The patient’s anemia responded well to the treatment and his symptoms began to improve after 2 weeks of the treatment. Lifelong parenteral vitamin B-12 treatment has anomaplanned. Gastroscopic examination resulted in a polyp of cardia that was impossible to remove endoscopically for technical reasons. Histopathological examination of the biopsy specimen showed in situ adenocarcinoma and total gastrectomy was performed. Histopathological examination of the gastrectomy material showed foveolar highgrade dysplasia, elevated p53 expression with p53-positive staining, expansive intestinal metaplasia (complete type) in cardiac mucosa, low-grade chronic gastritis, and patching intestinal metaplasia in the antrum (Figure 3). 

PS is a rare congenital disease characterized by unilateral agenesis of the pectoral muscles, various ipsilateral deformities of the upper extremities, and malformations of the anterior chest wall. Genetic and teratogenic factors might play a role in its etiology. Clinical manifestations are variable. The incidence of PS varies from 1/7,000 to 1/100,000. The right side of the body and males are more commonly affected. PS is an autosomal dominant transition trait; nonetheless, some researchers reported that there is not a genetic association. Many cases are sporadic. Hypoplasia of the subclavian artery, and the vertebral arteries and their branches due to a momentary interruption or reduction in embryonic development is observed in the syndrome [1,2]. Absence of costal cartilage or the ribs, diaphragm hernias, heart anomalies such as dextrocardia, central nervous system and genitourinary system anomalies, and vertebral anomalies are also associated with PS [3]. An autoimmune disease with dysplastic mucosa of the stomach in association with this congenital anomaly has, to date, not been reported. 

PS can be present with various deformities of the thoracic wall and upper extremities, skin pathologies, and cardiac anomalies. Such malformations are associated with morbidity and limited social activity. Congenital anoma lies of the pectoral muscles cause little or no functional deficit in normal daily activities. The presented patient did not have any complaints about his extremities and had no functional deficits. Various surgical techniques have been described for the repair of chest wall defects in PS [4,5,6]. The presented patient admitted to our hospital with symptoms of anemia. Macrocytic anemia, very low vitamin B-12 level, low reticulocyte index, splenomegaly, and the presence of anti-parietal and anti-intrinsic factor antibodies were indicative of the diagnosis of pernicious anemia. 

Pernicious anemia is an autoimmune, heritable multifactorial disorder. The presence of a hemoglobin concentration <13 g dL–1 in men and <12 g dL–1 in women, mean corpuscular volume ≥100 fL, and low vitamin B-12 level, together with the concomitant presence of atrophic gastritis and intrinsic factor deficiency are the findings reminding pernicious anemia. The presence of intrinsic factor serum antibodies and hypergastrinemia can support the diagnosis of pernicious anemia; however, the absence of these findings does not exclude the diagnosis. It was reported that 10%-20% of patients with pernicious anemia also have atrophic gastritis of the antrum, with gastrin cell reduction and normal serum gastrin levels [7,8,9]. The sensitivity of intrinsic factor antibodies in the diagnosis of pernicious anemia is 50%-70% [10]. 

Pernicious anemia with elevated serum levels of parietal cell antibodies occurs in 60%-90% of cases and is considered a sequela of autoimmune atrophic gastritis (AMAG). Chronic atrophic gastritis with intestinal metaplasia, gastric polyps, and gastric dysplasia is frequently observed in patients with pernicious anemia. AMAG is associated with an increased risk of gastric cancer. Among patients with AMAG, the incidence of gastric carcinoma and carcinoids is 1%-3% and 1%-7%, respectively [9,10,11,12]. Armbrect et al. recommend performing endoscopy with multiple biopsies at least once at the time of diagnosis in all patients with pernicious anemia. They also favor removing polyps and re-examining patients with polyps [12]. As such, we performed gastroscopy in the presented patient following the diagnosis of pernicious anemia. Abnormal physical examination findings of the chest wall led us to perform thoracic CT and the patient was subsequently diagnosed with PS. Neoplastic pathologies are sometimes seen with PS. Wilms tumor, carcinoma of the breast, lung cancer, and neuroblastoma have been reported in some PS patients [13,14,15]. PS has been reported in association with hematological malignancies, such as acute leukemias and chronic granulocytic leukemia, and lymphomas [16]; however, to the best of our knowledge gastric neoplasia in a PS patient has not been previously reported. 

The etiology of PS remains unknown. Several etiopathogenic mechanisms underlying PS have been hypothesized, including vascular defect due to an insult during early embryologic stages, paradominant inheritance, and the presence of an autosomal lethal gene surviving via mosaicism. To the best of our knowledge this is the first report of an autoimmune disorder in a patient with PS. Additionally, there are no reports of gastric malignancy in PS. Due to a possible predisposition to autoimmune diseases, such as pernicious anemia, clinicians should consider autoimmune disorders and malignancies while examining patients with PS. Informed consent was obtained. 

**Conflict of Interest Statement**

The authors declare that they have no conflicts of interest that could be perceived as having influenced the impartiality of the materials presented. 

**Funding**

The present study received no grant from a funding agency in the public, commercial, or a profit sector.

## Figures and Tables

**Figure 1:**
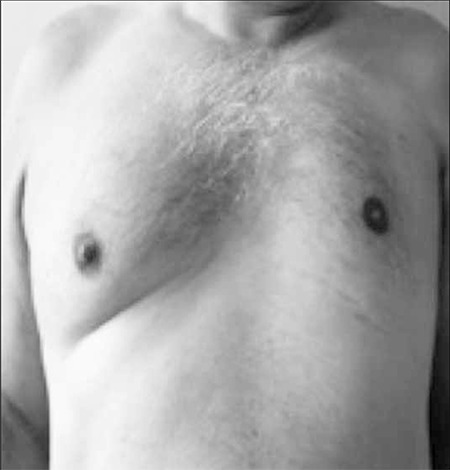
Asymmetric appearance of the nipples.

**Figure 2:**
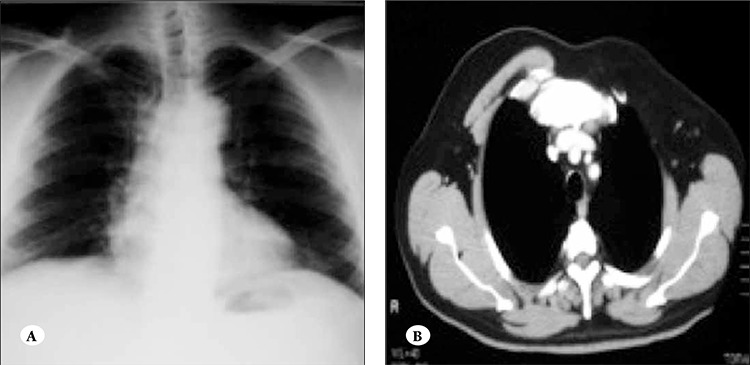
(A) Chest X-ray shows hyperlucency on the left side of the chest. (B) CT image shows agenesis of the left pectoralis major muscle.

**Figure 3:**
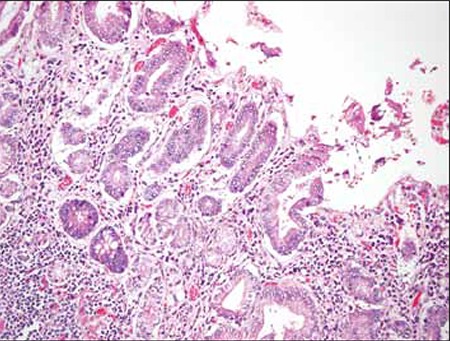
High-grade dysplasia in the cardiac mucosa (40x magnification with hematoxylin and eosin stain).
